# Characterisation of Lipid Changes in Ethylene-Promoted Senescence and Its Retardation by Suppression of Phospholipase Dδ in *Arabidopsis* Leaves

**DOI:** 10.3389/fpls.2015.01045

**Published:** 2015-11-30

**Authors:** Yanxia Jia, Weiqi Li

**Affiliations:** ^1^Germplasm Bank of Wild Species, Kunming Institute of Botany, Chinese Academy of SciencesKunming, China; ^2^Key Laboratory for Plant Diversity and Biogeography of East Asia, Kunming Institute of Botany, Chinese Academy of SciencesKunming, China

**Keywords:** *Arabidopsis* leaf senescence, ethylene, lipidomics, membrane lipids, phospholipase Dδ

## Abstract

Ethylene and abscisic acid (ABA) both accelerate senescence of detached *Arabidopsis* leaves. We previously showed that suppression of Phospholipase Dδ (PLDδ) retarded ABA-promoted senescence. Here, we report that ethylene-promoted senescence is retarded in detached leaves lacking *PLDδ*. We further used lipidomics to comparatively profile the molecular species of membrane lipids between wild-type and PLDδ-knockout (PLDδ-KO) *Arabidopsis* during ethylene-promoted senescence. Lipid profiling revealed that ethylene caused a decrease in all lipids levels, except phosphatidic acid (PA), caused increases in the ratios of digalactosyl diglyceride/monogalactosyl diglyceride (MGDG) and phosphatidylcholine (PC)/phosphatidylethanolamine (PE), and caused degradation of plastidic lipids before that of extraplastidic lipids in wild-type plants. The accelerated degradation of plastidic lipids during ethylene-promoted senescence in wild-type plants was attenuated in PLDδ-KO plants. No obvious differences in substrate and product of PLDδ-catalyzed phospholipid hydrolysis were detected between wild-type and PLDδ-KO plants, which indicated that the retardation of ethylene-promoted senescence by suppressing PLDδ might not be related to the role of PLDδ in catalyzing phospholipid degradation. In contrast, higher plastidic lipid content, especially of MGDG, in PLDδ-KO plants was crucial for maintaining photosynthetic activity. The lower relative content of PA and higher PC/PE ratio in PLDδ-KO plants might contribute to maintaining cell membrane integrity. The integrity of the cell membrane in PLDδ-KO plants facilitated maintenance of the membrane function and of the proteins associated with the membrane. Taking these findings together, higher plastidic lipid content and the integrity of the cell membrane in PLDδ-KO plants might contribute to the retardation of ethylene-promoted senescence by the suppression of PLDδ.

## Introduction

Leaf senescence, the final stage of leaf development, is a genetically regulated, highly ordered process by which plants mobilize and recycle nutrients from leaves to other plant parts, such as seeds, storage organs, or developing leaves and flowers (Lim et al., 2007). Chlorophyll degradation is the first visible symptom of senescence, and chloroplast membrane degradation, which parallels a loss in photosynthetic activity, was shown to occur before degradation of the membranes of other organelles (Woolhouse, 1984). Chloroplast membranes, especially the plastidic membrane, are believed to be highly vulnerable to heat-stress-associated damage (Woolhouse, 1984; Wanner et al., 1991), and the damage of these membranes is an event that occurs at an early stage during leaf senescence (Guo and Gan, 2005). The plastidic membrane consists mainly of four lipids: monogalactosyl diglyceride (MGDG), digalactosyl diglyceride (DGDG), phosphatidylglycerol (PG), and sulfoquinovosyl diacylglycerol (SQDG; Devaiah et al., 2006), with MGDG and DGDG comprising 70–80% of the plastidic lipid matrix associated with photosynthetic membranes. The cytosolic leaflet of the outer envelope membrane also contains a few phosphatidylcholine (PC; Devaiah et al., 2006).

Membranes of extraplastidic organelles mainly consist of phospholipids, and only a fraction of the extraplastidic membranes contain very small amount DGDG (Devaiah et al., 2006). The degradation of phospholipids is mediated by several enzyme cascades initiated by various phospholipases, including phospholipases A, C, and D. Phospholipase D (PLD) hydrolyzes phospholipids into phosphatidic acid (PA) and head group, and it has 12 members in *Arabidopsis* (Qin and Wang, 2002). The suppression of major PLD, PLDα1, retards abscisic acid (ABA)- or ethylene-promoted senescence (Fan et al., 1997). Phospholipid Dδ (PLDδ), one of most abundant PLDs, has several properties that distinguish it from other PLDs (Wang and Wang, 2001). PLDδ is activated by oleic acid and is tightly associated with the plasma membrane and microtubule (MT) cytoskeleton (Gardiner et al., 2001; Dhonukshe et al., 2003; Zhang et al., 2012). Analyses of PLDδ-altered *Arabidopsis* suggest that PLDδ positively regulates plant tolerance to stresses such as freezing (Li et al., 2004, 2008) and ultraviolet irradiation (Zhang et al., 2003). Our previous study found that the suppression of PLDδ retards ABA-promoted senescence through attenuating PA production (Jia et al., 2013). However, whether PLDδ functions in ethylene-promoted senescence is unknown so far.

Ethylene is considered to be a major hormonal regulator of senescence in most plant organs, including leaf, cotyledon, and petal (Grbic and Bleecker, 1995). It promotes senescence through the enhancement of various lipid catabolic processes (Baardseth and Vonelbe, 1989), and then the lipid metabolism enhances senescence through the regulation of ethylene production and/or action (Halevy et al., 1996). It is noted that the ethylene-mediated increase in membrane permeability in senescing *Tradescantia* correlated temporally with a reduction in the tissue levels of phospholipids (Suttle and Kende, 1980). The decline of phospholipid content is shown to result in the loss of membrane integrity and physical changes in plant membrane lipids during senescence, which greatly increased the permeability of lipid bilayers (Wanner et al., 1991; Cheour et al., 1992). Given that PLDδ is one of major lipolytic enzymes, whether it has a role in the lipid changes during ethylene promoted senescence remains to test.

In the present study, we compared the ethylene-promoted leaf senescence between Wassilewskija (WS) ecotype and PLDδ-knockout mutant *Arabidopsis* and found that suppression of PLDδ retarded ethylene-promoted senescence. Profiling the changes of molecular lipid species by using electrospray ionization tandem mass spectrometry (ESI-MS/MS) in WS and PLDδ-KO detached leaves revealed how lipid changes and provided insight into the function of PLDδ in ethylene-promoted senescence.

## Materials and Methods

### Plant Materials, Growth Conditions, and Hormone Treatments

A PLDδ-knockout mutant was previously isolated from *Arabidopsis* Wassilewskija ecotype (WS). The loss of PLDδ was confirmed by the absence of its transcript, protein and activity (Li et al., 2008).

Two *Arabidopsis* genotypes were grown in water in a controlled growth chamber at 23°C (day) and 19°C (night) and 60% relative humidity under a 12 h photoperiod, with fluorescent lighting at 120 μmol m^-2^ sec^-1^. Fully expanded leaves of the same age were collected from approximately 6-week-old plants of the two genotypes of *Arabidopsis*; the detached leaves were rinsed briefly with sterile water and placed with the adaxial side up in Petri dishes containing 50 μM ethephon (Sigma, C0143). We chose ethephon rather than ethylene for incubation of the detached leaves because it is easier to control, but has an identical effect on the detached leaves. The leaves were incubated at 23°C under a 12 h photoperiod and light at 120 μmol m^-2^ sec^-1^.

### Measurements of Chlorophyll Content, Photosynthetic Activity and Cell Death

Chlorophyll was extracted incubation of leaves after incubation with ethephon in, 80% acetone. Chlorophyll content was determined spectrophotometrically at 663 and 646 nm as described previously (Craftsbrandner et al., 1984). Chlorophyll fluorescence was analyzed using an imaging chlorophyll fluorometer, MAXI-Imaging Pulse-Amplitude (PAM; Walz, Germany; Bonfig et al., 2006). The maximal quantum yield of photosystem II (PS II; *F*_v_/*F*_m_) photochemistry was measured after adaptation to complete darkness for 20 min.

Cell death, indicated by loss of plasma membrane integrity, was quantified spectrophotometrically by Evans blue staining of detached leaves, using a previously described method with minor modifications (Rea et al., 2004). Briefly, detached leaves were incubated with 0.1% (w/v) Evans blue for 2 h with shaking, and then washed extensively to remove unbound dye. The leaves were ground into powder in liquid nitrogen. The tissue powder was incubated with 50% (v/v) methanol and 1% (w/v) SDS at 60°C for 30 min, and then centrifuged. For a control measurement of 100% cell death, the leaves were heated at 100°C for 5 min. Absorbance was measured at 600 nm.

### Lipid Extraction and Analysis

The processes of lipid extraction, ESI-MS/MS analysis and quantification were performed in accordance with a protocol from Welti et al. (2002). Data processing was performed as previously described. The lipids in each class were quantified by comparison with two internal standards of the class. Five replicates of each treatment for each genotype were analyzed. The *Q* test was performed on the total amount of lipid in each head-group class, and data from discordant samples was removed (Devaiah et al., 2006).

### Data Analysis

Statistical analysis was performed using Origin 7.0 (Origin Lab Corporation, Northampton, MA, USA). For all quantitative measurements in this study, five replicates from each sampling time were analyzed. The data were subjected to one-way ANOVA analysis (duncan’s multiple range test) of variance with SPSS 16.0.

## Results

### Suppression of PLDδ Retarded Ethylene-promoted Senescence

**FIGURE 1:**
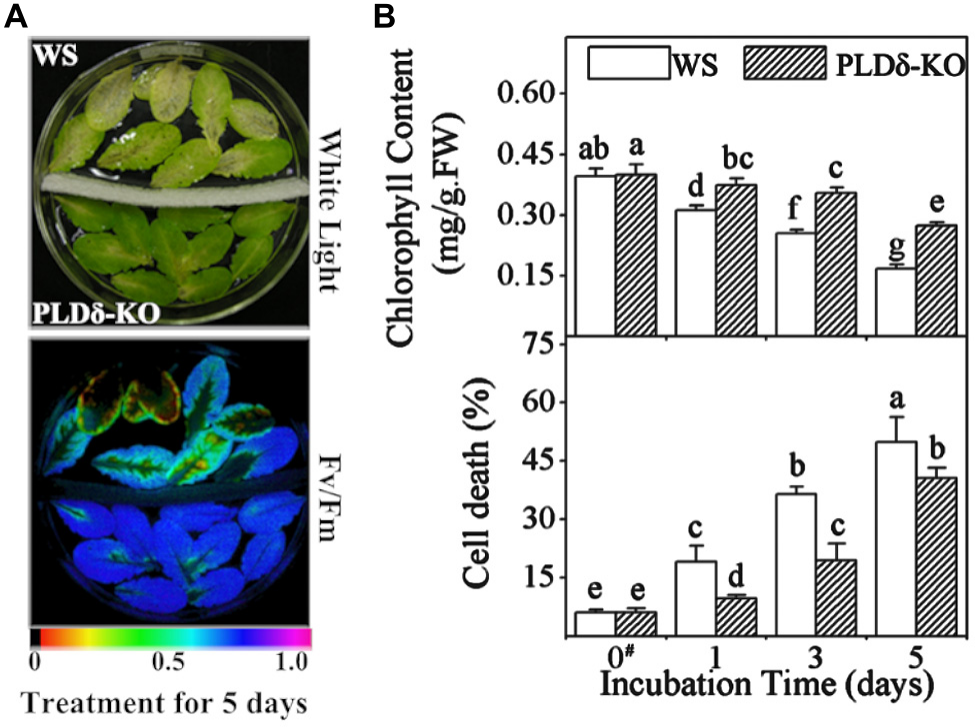
**Retardation of abscisic acid (ABA)-promoted senescence was compared between wassilewskija (WS) and PLDδ-KO detached leaves.** Leaves detached from WS and PLDδ-KO plants were treated with sterile 50 μM ethephon for 5 days. **(A)** Yellow coloration (left) or low *F*v/*F*m values for variable fluorescence (right) indicated senescence. The color bar on the bottom indicates *F*v/*F*m values. **(B)** Chlorophyll content (top) and cell death rate (bottom) of leaves from WS and PLDδ-KO plants. FW, fresh weight. Values are means ± SD (*n* = 5). Values with different letters are significantly different (*p* < 0.05). Annotation: data of “#” column is from Jia et al. (2013).

Leaves detached from WS plants started yellowing 1 day after treatment and turned almost completely yellow 5 days after incubation in 50 μM ethephon under light. In contrast, most parts of the PLDδ-KO leaves were still green after the 5-day ethylene treatment, which indicated a much slower senescence process in the PLDδ-deficient leaves (**Figure 1A**, top). Consistent with the visible yellowing, the photochemical quantum efficiency of the photosystem II (PS II) reaction center (*F*_v_/*F*_m_) in ethylene-treated leaves was much lower in WS plants than in PLDδ-KO mutants (**Figure 1A**, bottom). Measurements of chlorophyll content showed that chlorophyll was lost more quickly from WS leaves, diminishing by 37% after 5 days, whereas PLDδ-KO mutant leaves lost just 20% of their chlorophyll content upon treatment with ethylene (**Figure 1B**, top). Data on cell viability showed that the rate of cell death was significantly higher in the WS leaves during the ethylene-promoted senescence process, as measured by Evans blue staining (**Figure 1B**, bottom). These results indicate that the suppression of PLDδ retarded, to some extent, ethylene-promoted senescence.

### Large Changes in Lipid Profiles Occurred during Ethylene-promoted Senescence

Metabolism of membrane lipids is one of several biochemical manifestations of cellular senescence (Thompson et al., 1998). Since the above results suggest that PLDδ is involved in ethylene-promoted senescence, combined with the participation of PLDδ in the metabolism of membrane lipids, we performed ESI-MS/MS analysis to determine whether the degradation of membrane lipids is affected by the suppression of PLDδ during ethylene-promoted senescence. ESI-MS/MS allowed the identification of alterations in >120 diverse polar glycerolipids, including six head-group classes of phospholipids [PC, phosphatidylethanolamine (PE), phosphatidylinositol (PI), phosphatidylserine (PS), PA, and PG] and two head-group classes of galactolipids (MGDG and DGDG; **Table 1**). Each molecular species was identified in terms of the total numbers of acyl carbon atoms and double bonds (Welti et al., 2002).

**Table 1:** Total lipids in leaves of Wassilewskija (WS) and PLDδ-KO plants during ethylene-promoted senescence.

Lipids	Genotypes	Lipids/dry weight (nmol/mg)			RC (%)	
		Day 0^#^	Day 3	Day 5	Day 3	Day 5
PG	WS	12.86 ± 1.89^a^	10.82 ± 2.92^a^	2.14 ± 0.72^c^	-	-83.4
	PLDδ-KO	12.67 ± 4.97^a^	6.73 ± 1.51^b^	4.56 ± 1.76^b^	-46.9	-64.0
PC	WS	16.73 ± 1.56^a^	17.97 ± 6.90^a^	9.25 ± 2.52^b^	-	-44.7
	PLDδ-KO	15.21 ± 4.66ˆab	13.82 ± 3.36ˆab	10.93 ± 2.00ˆab	-	-
PE	WS	9.86 ± 1.09^a^	8.53 ± 3.27ˆab	5.28 ± 1.85ˆbc	-	-46.5
	PLDδ-KO	9.17 ± 2.81^a^	6.43 ± 1.67ˆab	5.13 ± 1.43^c^	-	-44.1
PI	WS	2.38 ± 0.56ˆab	2.90 ± 0.80^a^	1.62 ± 0.87^b^	-	
	PLDδ-KO	2.55 ± 0.52ˆab	3.14 ± 1.00^a^	2.05 ± 0.53ˆab	-	-
PA	WS	0.07 ± 0.05^a^	0.10 ± 0.01^a^	0.07 ± 0.03^a^	-	-
	PLDδ-KO	0.09 ± 0.06^a^	0.09 ± 0.02^a^	0.08 ± 0.02^a^	-	-
PS	WS	0.35 ± 0.06^a^	0.12 ± 0.11^b^	0.33 ± 0.22ˆab	-65.7	-
	PLDδ-KO	0.20 ± 0.06ˆab	0.14 ± 0.07^b^	0.36 ± 0.08^a^	-	-
MGDG	WS	235.55 ± 17.65^a^	175.83 ± 19.04^b^	76.69 ± 16.18^d^	-25.4	-67.4
	PLDδ-KO	217.78 ± 25.85^a^	187.69 ± 43.08^b^	103.67 ± 13.18^c^	-13.8	-52.4
DGDG	WS	31.73 ± 4.78^a^	25.22 ± 2.07^c^	14.80 ± 1.25^e^	-20.5	-53.4
	PLDδ-KO	31.85 ± 6.85^a^	28.07 ± 1.83^b^	16.43 ± 1.59^d^	-11.9	-48.4
				
		**Total lipids/dry weight (nmol/mg)**				
				
Total lipids	WS	302.20 ± 28.25^a^	249.05 ± 8.51^b^	94.53 ± 4.60^d^	-17.6	-68.7
	PLDδ-KO	283.52 ± 38.85^a^	252.81 ± 4.85ˆab	139.84 ± 4.99^c^	-	-50.7

*The relative change (RC) in the levels of lipids from days 0 to 3 and 5 is the percentage value for the significant difference between the values at day 0 and days 3 and 5 over the value at day 0. Values in the same lipid molecular species with different letters are significantly different (p < 0.05). Values are means ± SD (n = 5). Annotation: data of “#” column is from Jia et al. (2013).*

**FIGURE 2:**
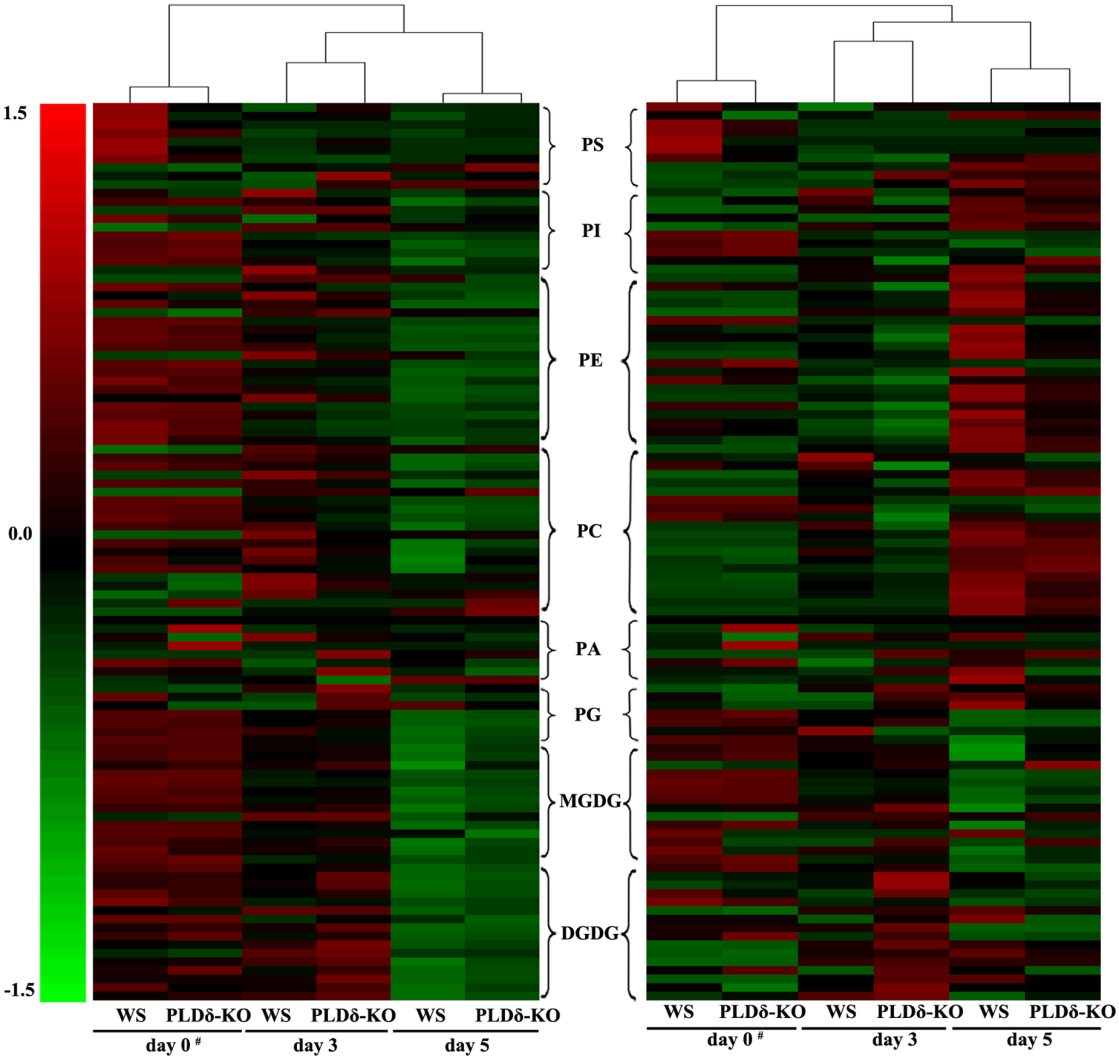
**Hierarchical clustering analysis of lipid molecular species during ethylene-promoted senescence.** Absolute (nmol/mg dry weight) **(left)** and relative levels (mol%) of lipid molecular species **(right)**. The color of each bar represents the abundance of the corresponding lipid species. Expression is shown as the relative change from the mean center of each lipid species. Lipid species in the indicated lipid classes were organized using class (as indicated), total acyl carbons (in ascending order within a class), and total double bonds (in ascending order within a class and number of total acyl carbons). Annotation: data of “#” column is from Jia et al. (2013).

As an overview, most lipid species changed dramatically in terms of both their level (absolute value; **Figure 2**, left) and the composition (relative value; **Figure 2**, right) during ethylene-promoted senescence in the leaves of both genotypes of *Arabidopsis*. The levels of most lipids declined in both WS and PLDδ-KO leaves, although there were some differences in the profiles of membrane lipids between plants of the two genotypes during ethylene-promoted senescence (**Figure 2**). Clustering of the lipid contents of leaves in ethylene-promoted senescence suggested that the ethylene treatment was the main factor inducing the degradation of membrane lipids. The differences between WS and PLDδ-KO leaves subjected to ethylene treatment were greater than those between WS and PLDδ-KO leaves without such treatment (**Figure 2**). These results suggest that ethylene treatment affected lipid degradation, and that PLDδ participated in this lipid degradation during ethylene-promoted senescence.

### The Degradation of Plastidic Lipids Occurred before that of Extraplastidic Lipids during Ethylene-promoted Senescence

During ethylene-promoted senescence in WS leaves, the levels of leaf membrane lipids decreased significantly (**Table 1**). After ethylene treatment for 5 days, we found that the level of total lipids decreased by 68.7%, from 302.20 nmol/mg (non-senescent leaves, NS) to 94.53 nmol/mg (leaves treated with ethylene for 5 days). The levels of PG, PC, PE, DGDG, and MGDG all decreased significantly, but the abundances of PA, PI, and PS remained unchanged. As shown in **Table 1**, after ethylene-promoted senescence for 3 days, the levels of MGDG and DGDG, two main classes of plastidic lipid, decreased significantly compared with those in untreated leaves, whereas the levels of the main extraplastidic lipids (PC and PE) decreased significantly only after ethylene treatment for 5 days. These findings imply that plastidic lipids might be degraded before extraplastidic lipids.

To compare lipid degradation between plastidic and extraplastidic membranes further, the changes in the levels of molecular species of PG were analyzed. In *Arabidopsis*, PG includes four molecular species, namely, PG 34:1 (total carbon number:double bond number), 34:2, 34:3, and 34:4 (Welti et al., 2002). PG 34:4, which contains a 16:1 acyl chain at the *sn*-2 position, is part of the plastidic membrane, whereas both PG 34:1 and 34:2 are extraplastidic lipids. Of the two molecules that correspond to PG 34:3, one contains a 16:1 acyl chain which is part of the plastidic membrane, whereas the other is extraplastidic (Marechal et al., 1997). During ethylene-promoted senescence in WS leaves, the level of PG 34:4 had decreased by 33.2% at day 3, whereas the levels of the other three PG molecular species, PG 34:3, 34:2, and 34:1, showed no significant decline after ethylene treatment for 3 days (**Table 2**). These results indicate that the degradation of plastidic membrane lipids occurred before that of extraplastidic lipids during ethylene-promoted senescence.

**Table 2:** Levels of PG molecular species in leaves of WS and PLDδ-KO plants during ethylene-promoted senescence.

PG	Genotypes	Lipids/dry weight (ng/mg)			RC (%)	
		Day 0^#^	Day 3	Day 5	Day 3	Day 5
34:1	WS	0.65 ± 0.19^a^	0.37 ± 0.32ˆab	0.05 ± 0.06^b^		-91.7
	PLDδ-KO	0.69 ± 0.33^a^	0.42 ± 0.49ˆab	0.10 ± 0.14^b^		-86.0
34:2	WS	0.98 ± 0.32^a^	0.57 ± 0.36^a^	0.18 ± 0.19^b^		-81.4
	PLDδ-KO	0.93 ± 0.15^a^	0.76 ± 0.41^a^	0.24 ± 0.12^b^		-74.3
34:3	WS	2.80 ± 0.17^a^	2.69 ± 0.23^a^	0.79 ± 0.27^c^		-71.9
	PLDδ-KO	3.07 ± 0.47^a^	2.97 ± 0.01^a^	1.19 ± 0.13^b^		-61.2
34:4	WS	8.18 ± 1.56^a^	5.46 ± 1.37^b^	1.35 ± 0.62^c^	-33.3	-83.6
	PLDδ-KO	7.84 ± 1.67^a^	4.66 ± 1.90^b^	2.79 ± 1.20^b^	-40.5	-64.4

*The RC in lipids from day 0 to day 5 is the percentage value for the significant difference between the values at day 0 and day 3 and 5 over the value at day 0. Values in the same lipid molecular species with different letters are significantly different (p < 0.05). Values are means ± SD (n = 5). Annotation: data of “#” column is from Jia et al. (2013).*

### Suppression of PLDδ Attenuated the Decrease in Levels of Plastidic Lipids during Ethylene-promoted Senescence

During ethylene-promoted senescence, the amount of total lipids declined by 50.7% in PLDδ-KO plants (**Table 1**). Most of this decrease could be attributed to a decrease in plastidic lipids. For example, the level of MGDG in PLDδ-KO leaves decreased by 52.4% (from 217.78 to 103.67 nmol/mg), the level of DGDG decreased by 48.4% (from 31.85 to 16.43 nmol/mg), and the level of PG decreased by 64.0% (from 12.67 to 4.56 nmol/mg). The levels of both total lipids and the main plastidic lipids (MGDG, DGDG) were significantly higher in PLDδ-KO leaves than in WS leaves, whereas no differences in the levels of PC, PE, PI, PA, and PS were detected between the plants with different genotypes (**Table 1**). Upon further analysis of the content of molecular species of each membrane lipid, we found that the contents of molecular species MGDG 34:2, 34:5, 34:6, and 36:6 as well as DGDG 36:6 in PLDδ-KO detached leaves were much higher than that in WS detached leaves (**Table 3**). Furthermore, the levels of the plastidic lipids PG 34:4 and 34:3 were higher in PLDδ-KO leaves than that in WS leaves. The extraplastidic lipids PG 34:2 and 34:1 in PLDδ-KO leaves showed no clear difference compared with those in WS leaves (**Table 2**). The lipids that are largely synthesized and localized in plastids, PG 34:4, MGDG, and DGDG, were previously shown to be the most abundant in leaves (Devaiah et al., 2006). Plastidic lipids have also been shown to have a direct role in photosynthesis (Dormann and Benning, 2002). We also found that the main MGDG species 34:6 and 36:6 significantly decreased at day 3 of ethylene treatment, whereas the level of DGDG 34:6 increased obviously. These results indicated that these head group remodeling of galactolipids might contribute to maintenance of photosynthesis at early stage of leaf senescence. Combining these findings with our results, it is conceivable that the attenuated degradation of plastidic lipids might have contributed to higher photosynthetic activity in PLDδ-KO leaves during ethylene-promoted senescence, and the retardation of ethylene-promoted senescence by the suppression of PLDδ was a consequence of qualitative changes in plastidic lipids.

**Table 3:** Levels of major lipid molecular species in leaves of WS and PLDδ-KO plants during ethylene-promoted senescence.

Galactolipids major lipids species	Genotypes	Lipids/dry weight (nmol/mg)			RC (%)	
		Day 0	Day 3	Day 5	Day 3	Day 5
DGDG 34:6	WS	2.51 ± 0.41^b^	3.36 ± 0.54^a^	1.53 ± 0.59^c^	33.9	-39.0
	PLDδ-KO	2.26 ± 0.61^b^	3.29 ± 0.59^a^	1.85 ± 0.97^bc^	45.6	-
DGDG 34:5	WS	0.36 ± 0.07^a^	0.13 ± 0.04^b^	0.04 ± 0.02^c^	-63.9	-88.9
	PLDδ-KO	0.29 ± 0.07^a^	0.15 ± 0.03^b^	0.07 ± 0.04^c^	-48.3	-75.9
DGDG 34:3	WS	6.47 ± 1.10^a^	5.67 ± 2.35^a^	2.41 ± 1.07^b^	-	-62.8
	PLDδ-KO	6.80 ± 1.14^a^	7.75 ± 3.67^a^	3.15 ± 1.42^b^	-	-53.7
DGDG 34:2	WS	0.53 ± 0.05^a^	0.44 ± 0.23^a^	0.18 ± 0.07^b^	-	-66.0
	PLDδ-KO	0.54 ± 0.16^a^	0.65 ± 0.47ˆab	0.22 ± 0.09^b^	-	-59.3
DGDG 36:6	WS	19.98 ± 3.17^a^	23.60 ± 8.68^a^	10.87 ± 2.42^c^	-	-45.6
	PLDδ-KO	20.26 ± 6.08ˆab	25.52 ± 5.63^a^	16.96 ± 5.05^b^	-	-
DGDG 36:5	WS	0.63 ± 0.11^a^	1.06 ± 0.50^a^	0.56 ± 0.17^a^	-	-
	PLDδ-KO	0.53 ± 0.17^a^	1.14 ± 0.60^a^	0.60 ± 0.21^a^	-	-
MGDG 34:6	WS	218.94 ± 43.22^a^	139.36 ± 46.31^b^	68.55 ± 14.48^d^	-36.4	-68.7
	PLDδ-KO	207.77 ± 52.62^a^	161.64 ± 33.84^b^	90.66 ± 11.24^c^	-22.2	-56.4
MGDG 34:5	WS	7.89 ± 1.50^a^	1.92 ± 0.86^c^	0.26 ± 0.09^e^	-75.7	-96.7
	PLDδ-KO	6.76 ± 2.15^a^	2.51 ± 1.19^b^	0.66 ± 0.05^d^	-62.9	-90.2
MGDG 34:4	WS	2.91 ± 0.65^a^	1.24 ± 0.48^b^	0.22 ± 0.09^c^	-57.4	-92.4
	PLDδ-KO	2.90 ± 0.87^a^	1.49 ± 0.62^b^	0.47 ± 0.27^c^	-48.6	-83.8
MGDG 34:3	WS	1.69 ± 0.34^a^	1.64 ± 0.71^a^	0.62 ± 0.27^b^	-	-63.3
	PLDδ-KO	1.86 ± 0.66^a^	1.93 ± 0.80^a^	1.36 ± 0.75^ab^	-	-
MGDG 34:2	WS	0.33 ± 0.08^a^	0.22 ± 0.09^a^	0.54 ± 0.38^a^	-	-
	PLDδ-KO	0.34 ± 0.11^a^	0.25 ± 0.12ˆab	0.13 ± 0.07^b^	-	-61.8
MGDG 36:6	WS	22.74 ± 2.93^a^	15.79 ± 5.58^bc^	5.48 ± 2.33^d^	-30.6	-75.9
	PLDδ-KO	23.15 ± 6.14^a^	17.90 ± 6.00^ab^	9.30 ± 2.69^c^	-	-59.8
MGDG 36:5	WS	0.68 ± 0.19^b^	1.28 ± 0.51^a^	0.43 ± 0.15^c^	88.2	-36.8
	PLDδ-KO	0.60 ± 0.17^b^	1.31 ± 0.48^a^	0.77 ± 0.44ˆab^c^	118.3	-
MGDG 36:4	WS	0.42 ± 0.06^a^	0.35 ± 0.15^a^	0.11 ± 0.04^b^	-	-73.8
	PLDδ-KO	0.44 ± 0.13^a^	0.41 ± 0.18^a^	0.20 ± 0.09^b^	-	-54.6

*The RC in the levels of lipids from days 0 to 3 and day 5 is the percentage value for the significant difference between the values at day 0 and days 3 and 5 over the value at day 0. Values in the same lipid molecular species with different letters are significantly different (p < 0.05). Values are means ± SD (n = 5).*

### Changes in the Composition of Lipid Classes during Ethylene-promoted Senescence

For the analysis of the relative contents of membrane lipids, for which the data are expressed as mol% lipids, we found that the most important changes concerned the two galactolipids in WS plants after ethylene treatment for 5 days. The MGDG percentage decreased from 77.55% (NS) to 63.70% (leaves treated with ethylene for 5 days). In contrast, the DGDG percentage increased from 9.70% (NS) to 14.22% (leaves treated with ethylene for 5 days). The PG percentage showed a slight decrease in WS plants. In addition, the relative percentages of the non-chloroplastic PE, PC, PI, and PS, which are mainly located in the membranes of non-photosynthetic organelles such as the plasma membrane, endoplasmic reticulum, and mitochondria (Singh and Privett, 1970), increased during senescence, in keeping with the preferential destruction of chloroplast membranes. The ratio of galactolipids/phospholipids decreased from 11.02 to 5.41, which represent a decrease of 50.9% (**Table 4**). This decrease might have resulted from the degradation of 67.4% of the MGDG, which initially constituted 77.6% of total lipids (**Tables 1** and **4**). The ratio of DGDG/MGDG increased in both WS and PLDδ-KO leaves during ethylene-promoted senescence. The relative content of MGDG was clearly higher in PLDδ-KO plants than in WS plants, whereas the relative content of DGDG in PLDδ-KO plants resembled that in WS plants, which resulted in the ratio of DGDG/MGDG in PLDδ-KO detached leaves being significantly lower than that in WS plants after ethylene treatment for 5 days, namely, 0.19 and 0.22, respectively (**Table 4**). The higher relative content of MGDG in PLDδ-KO leaves might have contributed to stabilizing the ultrastructure, fluidity and permeability of the chloroplast membrane, which resulted in higher photosynthetic activity.

**Table 4:** Leaf membrane lipid composition in each head-group class and lipid ratio in WS and PLDδ-KO plants during ethylene-promoted senescence.

Lipid class	Genotype	Lipid (mol% of total lipid)		
		Day 0	Day 3	Day 5
PG	WS	3.92 ± 0.16^ab^	3.12 ± 0.72^c^	2.78 ± 0.28^c^
	PLDδ-KO	4.23 ± 0.33^a^	3.48 ± 0.82^bc^	3.15 ± 0.32^c^
PI	WS	0.78 ± 0.11^c^	1.13 ± 0.09^b^	2.03 ± 0.46^a^
	PLDδ-KO	0.82 ± 0.09^c^	1.23 ± 0.35ˆab	1.50 ± 0.38^a^
PS	WS	0.11 ± 0.02^b^	0.04 ± 0.02^c^	0.38 ± 0.13^a^
	PLDδ-KO	0.10 ± 0.05^b^	0.21 ± 0.24ˆab^c^	0.26 ± 0.01^a^
PA	WS	0.02 ± 0.01^b^	0.04 ± 0.02^b^	0.07 ± 0.02^a^
	PLDδ-KO	0.03 ± 0.01^b^	0.03 ± 0.01^b^	0.03 ± 0.01^b^
PC	WS	4.92 ± 0.55^bc^	7.30 ± 2.74^ab^	9.72 ± 1.70^a^
	PLDδ-KO	4.78 ± 0.42^c^	5.37 ± 0.91^b^	8.12 ± 1.79^a^
PE	WS	3.04 ± 0.32^a^	3.45 ± 0.23^a^	4.27 ± 1.14^a^
	PLDδ-KO	2.88 ± 0.26^a^	2.68 ± 0.54^a^	3.77 ± 1.10^a^
MGDG	WS	77.55 ± 1.22^a^	70.01 ± 1.86^c^	63.70 ± 3.48^d^
	PLDδ-KO	77.39 ± 1.66^a^	72.87 ± 1.40^b^	69.71 ± 3.18^bc^
DGDG	WS	9.70 ± 0.54^b^	13.86 ± 1.45^a^	14.22 ± 1.67^a^
	PLDδ-KO	10.08 ± 0.52^b^	15.36 ± 2.11^a^	13.41 ± 0.79^a^
		
		**Lipid ratio**		
		
PC/PE	WS	1.61 ± 0.07^c^	2.11 ± 0.12^b^	1.73 ± 0.14^c^
	PLDδ-KO	1.66 ± 0.04^c^	2.24 ± 0.37^ab^	2.39 ± 0.18^a^
DGDG/MGDG	WS	0.13 ± 0.01^c^	0.20 ± 0.03^ab^	0.22 ± 0.01^a^
	PLDδ-KO	0.13 ± 0.01^c^	0.21 ± 0.03^ab^	0.19 ± 0.01^b^
Galactolipids/Phospholipids	WS	11.02 ± 1.21^a^	9.50 ± 0.71^b^	5.41 ± 1.60^c^
	PLDδ-KO	11.24 ± 0.94^a^	9.41 ± 1.12^b^	6.64 ± 1.24^c^

*Values in the same lipid molecular species with different letters are significantly different (p < 0.05). Values are means ± SD (n = 5).*

### The Lower Relative Content of PA and Higher Ratio of PC/PE Might Contribute to the Retardation of Ethylene-promoted Senescence in PLDδ-KO Plant Leaves

**FIGURE 3:**
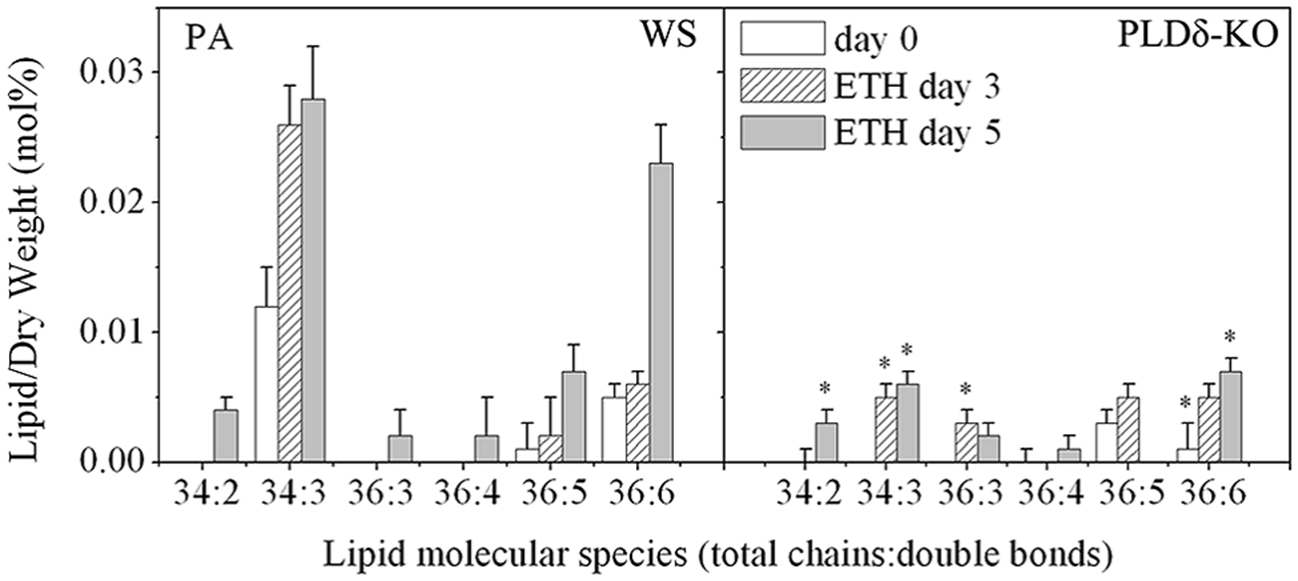
**Changes in the molecular species of PA in WS and PLDδ-KO plants during ethylene-promoted senescence.** “^∗^” indicates that the value is significantly different from that of the WS under the same conditions (*p* < 0.05). Values are means ± SD (*n* = 4 or 5).

To investigate how PLDδ functions in ethylene-promoted senescence, we analyzed the changes in the absolute level and relative content of PA under ethylene treatment in the two genotypes plants leaves. During ethylene-promoted senescence, no significant changes were detected in absolute levels of PA in either WS or PLDδ-KO plants. Upon analysis of the relative content of membrane lipids, we found that the relative content of PA increased 3.5-fold (from 0.02 to 0.07%) in WS plants, but remained unchanged in PLDδ-KO plants, which resulted in the relative content of PA in WS being much higher than that in PLDδ-KO plants after ethylene treatment for 5 days, especially for the molecular species PA 34:3, 36:3, and 36:6 (**Tables 1** and **3**; **Figure 3**). PA is a non-bilayer lipid and a potent promoter of the formation of the hexagonal phase and destabilization of the plasma membrane. For further assessment of the cell membrane stabilization of *Arabidopsis* during ethylene-promoted senescence, we calculated the PC/PE ratio in this process. This ratio in WS plants increased from 1.61 (NS) to 2.11 (leaves treated with ethylene for 3 days), and then decreased to the initial level of 1.73 (leaves treated with ethylene for 5 days). The PC/PE ratio in PLDδ-KO leaves increased constantly in the course of ethylene-promoted senescence, from 1.66 (NS) to 2.39 (leaves treated with ethylene for 5 days). In addition, the ratio of PC/PE in PLDδ-KO detached leaves was much higher than that in WS leaves after ethylene treatment for 5 days, namely, 2.39 and 1.73, respectively (**Table 4**). Our results indicate that the increase in the relative content of PA promoted destabilization of the plasma membrane; this may have led to the loss of membrane integrity and functions of membrane-associated proteins, thereby promoting senescence. Therefore, a reduction in the relative content of PA in PLDδ-KO leaves may have accounted for the higher ratio of PC/PE, which may have helped to maintain plasma membrane integrity and normal membrane protein function that eventually resulted in the retardation of ethylene-promoted senescence.

## Discussion

The senescence process takes place in a highly regulated manner and the cell constituents are dismantled via an ordered progression. Senescence affects both the plasma membrane and the intracellular membranes, which results in the loss of ionic and metabolite gradients that are essential for normal cell function (Fan et al., 1997; Thompson et al., 2000; He and Gan, 2002; Jia et al., 2013). With respect to organelle membranes, degradation occurs first at the plastidic membrane and last at the plasma membrane (Wanner et al., 1991). Many reports have indicated that ethylene accelerated the onset of membrane leakiness and phospholipid deterioration in leaves and petals (Suttle and Kende, 1980; Borochov et al., 1997). Our observations suggested that most membrane glycerolipids were degraded upon ethylene treatment, except for PS and PA, which were maintained unchanged, with the most extensive degradation occurring for MGDG, which plays a fundamental role in electron transfer between the antennae and cores of the photosystems (Siefermann-Harms et al., 1987; **Table 1**). With regard to the other plastidic lipids (DGDG and PG 34:4), ethylene treatment also caused noteworthy decreases in their levels. We also verified that the degradation of extraplastidic lipids was later to plastidic lipids, and the degradation of plastidic lipids paralleled a loss in photosynthetic activity and chlorophyll degradation, which is the first visible symptom of senescence. By the time leaf yellowing could be seen, extraplastidic lipid degradation had occurred. For example, the level of the main plastidic lipid MGDG declined by 25.4% when the chlorophyll content decreased to 69.8% of its initial level; extraplastidic lipids decreased until as the chlorophyll content decreased to 40.1% of its initial level in WS leaves after ethylene treatment for 5 days (**Tables 1–3** and **Figure 1**). Additionally, the degradation of lipids, in particular plastidic lipids, was accelerated during ABA-promoted senescence, and the degradation of plastidic lipids was earlier than extraplastidic lipids (Jia et al., 2013). Combined with the physiological observations, these results showed that degradation in levels of plastidic and extraplastidic lipids were correlated with the degree of leaf senescence during ABA- or ethylene-promoted senescence. One thing should be mentioned: given the drastic global lipid changes, the degradation of MGDG, PC, PE etc, we ignored the effects of PC in outer leaflet of chloroplast membranes and DGDG in the extraplastidic membranes on the concentration of plastidic lipids due to their small amount.

Both PC and DGDG have relatively large head groups, and tend to form a bilayer lipid phase. By contrast, PE and MGDG have small head groups involved in the formation of a non-bilayer lipid phase (Dormann and Benning, 2002; Welti et al., 2002). Adjustment of the molar PC/PE and DGDG/MGDG ratios is one of the most important approaches used by plants to respond to stress. The molar ratio of PC/PE tends to increase in plants under cold or hydration stress (Hazel and Williams, 1990; Welti et al., 2002). The DGDG/MGDG ratio increased significantly in salt-stressed jojoba leaves (Benrais et al., 1993) and in *Duboisia* leaves with reference to aging and senescence (Mishra et al., 1998). Earlier studies revealed that ethylene can facilitate both chemical and physical changes in the membrane lipids of senescing tissues, which presumably lead to the loss of intracellular compartmentalisation (Suttle and Kende, 1980; Thompson et al., 1982). During ethylene-promoted senescence, the PC/PE ratio increased (at day 3) and then decreased to its initial level (at day 5) in WS leaves, which indicated that the increase of the PC/PE ratio in early senescence stage might facilitate the maintenance of plasma membrane stability and function, and the decrease of the PC/PE ratio in late senescence stage might indicate cell membrane disintegration. Upon analysis of the relative contents of the two kinds of galactolipid, we found that the level of MGDG decreased, while that of DGDG increased, over the course of ethylene treatment, leading to an increase in the DGDG/MGDG ratio in both WS and PLDδ-KO plants (**Table 4**). This increase in the DGDG/MGDG ratio might help to maintain the chloroplast membrane in the bilayer conformation necessary for its biological functions, such as protein transport (Bruce, 1998) and photosystem activities, during leaf senescence (Dormann and Benning, 2002).

During senescence, the bulk of membrane phospholipids (i.e., PE, PC) were consumed by PLD, generating copious amounts of PA (Munnik, 2001; Munnik and Musgrave, 2001). PA can be further metabolized by PA phosphatase into diacylglycerol, a known activator of protein kinase C and a proposed enhancer of flower senescence (Borochov et al., 1997). PA was also shown to be a major intermediate during lipid metabolism, with both lipid degradation and lipid synthesis being regulated by the size of the PA pool (Li et al., 2009). PA is a non-bilayer lipid and a potent promoter of the formation of the hexagonal phase (Munnik, 2001). Suppression of PLDα1 and PLDδ partly blocks the formation of PA, and thus reduces lipid degradation, which may prolong membrane integrity and eventually retard ABA-promoted senescence (Fan et al., 1997; Jia et al., 2013). In the present study, the retardation of the senescence in PLDδ-KO leaves during ethylene treatment was indicated by delayed leaf yellowing, a higher level of chlorophyll, greater photosynthetic activity and a lower rate of cell death compared with those in WS leaves. PLDδ cleaves major membrane phospholipids, such as PE, PC, and PG, into PA and a free head group (Dyer et al., 1994). However, no significant differences in the substrate and product of the two-step transphosphatidylation reaction were detected between WS and PLDδ-KO plants during ethylene-promoted senescence, which indicated that the retardation of senescence by the suppression of PLDδ might not be related to the role of PLDδ in enzyme-catalyzed phospholipid hydrolysis.

Leaf senescence is accompanied by an early degradation of the cortical MT cytoskeleton in *Arabidopsis*, and the disruption of the MT network is affected by either repression or induction of microtubule-associated proteins (MAP; Keech et al., 2010). As a MAP in *Arabidopsis*, PLDδ acts as a bridge between the plasma membrane and MTs can thus convey external hormonal and environmental signal from plasma membrane to MT (Gardiner et al., 2001). It is possible that without PLDδ assistance, the signal of senescence through MT is blocked and therefore ethylene-promoted senescence is delayed in PLDδ-KO leaves.

## Conclusion

In this study, we have shown that the suppression of PLDδ effectively retarded ethylene-promoted senescence, indicated by higher chlorophyll content and photosynthetic activity, and a lower cell death rate. The profiles of membrane lipids suggested that the suppression of PLDδ attenuates plastidic lipid (PG 34:4, MGDG and DGDG) metabolism, while having no direct effect on the degradation of extraplastidic lipids. No obvious increase in product and decrease in substrate of the PLDδ-catalyzed phospholipid hydrolysis were detected, which indicated that the retardation of ethylene-promoted senescence in PLDδ-KO plants might not be related to the direct role of PLDδ in catalyzing phospholipids, and higher plastidic lipid (PG 34:4, MGDG and DGDG) content and PC/PE ratio in PLDδ-KO plants might contribute to maintenance of membrane integrity and function, and then help to retard senescence.

## Conflict of Interest Statement

The authors declare that the research was conducted in the absence of any commercial or financial relationships that could be construed as a potential conflict of interest.

## References

[B1] Baardseth P. Vonelbe J. H. (1989). Effect of ethylene, free fatty-acid, and some enzyme-systems on chlorophyll degradation. *J. Food Sci.* 54 1361–1363. 10.1111/j.1365-2621.1989.tb05993.x

[B2] Benrais L. Alpha M. J. Bahl J. Guillotsalomon T. Dubacq J. P. (1993). Lipid and protein contents of jojoba leaves in relation to salt adaptation. *Plant Physiol. Biochem.* 31 547–557.

[B3] Bonfig K. Schreiber U. Gabler A. Roitsch T. Berger S. (2006). Infection with virulent and avirulent *P. syringae* strains differentially affects photosynthesis and sink metabolism in *Arabidopsis* leaves. *Planta* 225 1–12. 10.1007/s00425-006-0303-3 16807755

[B4] Borochov A. Spiegelstein H. Philosoph-Hadas S. (1997). Ethylene and flower petal senescence: interrelationship with membrane lipid catabolism. *Physiol. Plant.* 100 606–612. 10.1034/j.1399-3054.1997.1000323.x

[B5] Bruce B. D. (1998). The role of lipids in plastid protein transport. *Plant Mol. Biol.* 38 223–246. 10.1023/A:1006094308805 9738969

[B6] Cheour F. Arul J. Makhlouf J. Willemot C. (1992). Delay of membrane lipid degradation by calcium treatment during cabbage leaf senescence. *Plant Physiol.* 100 1656–1660. 10.1104/pp.100.4.1656 16653181 PMC1075848

[B7] Craftsbrandner S. J. Below F. E. Harper J. E. Hageman R. H. (1984). Effects of pod removal on metabolism and senescence of nodulating and nonnodulating soybean isolines. *Plant Physiol.* 75 311–317. 16663617 10.1104/pp.75.2.311 PMC1066903

[B8] Devaiah S. P. Roth M. R. Baughman E. Li M. Tamura P. Jeannotte R. (2006). Quantitative profiling of polar glycerolipid species from organs of wild-type *Arabidopsis* and a PHOSPHOLIPASE Dα1 knockout mutant. *Phytochemistry* 67 1907–1924. 10.1016/j.phytochem.2006.06.005 16843506

[B9] Dhonukshe P. Laxalt A. M. Goedhart J. Gadella T. W. Munnik T. (2003). Phospholipase D activation correlates with microtubule reorganization in living plant cells. *Plant Cell* 15 2666–2679. 14508002 10.1105/tpc.014977 PMC280570

[B10] Dormann P. Benning C. (2002). Galactolipids rule in seed plants. *Trends Plant Sci.* 7 112–118. 10.1016/S1360-1385(01)02216-6 11906834

[B11] Dyer J. H. Ryu S. B. Wang X. (1994). Multiple forms of phospholipase D following germination and during leaf development of castor bean. *Plant Physiol.* 105 715–724. 12232238 10.1104/pp.105.2.715 PMC159413

[B12] Fan L. Zheng S. Wang X. (1997). Antisense suppression of phospholipase D alpha retards abscisic acid- and ethylene-promoted senescence of postharvest *Arabidopsis* leaves. *Plant Cell* 9 2183–2196. 10.2307/3870578 9437863 PMC157067

[B13] Gardiner J. C. Harper J. D. I. Weerakoon N. D. Collings D. A. Ritchie S. Gilroy S. (2001). A 90-kD phospholipase D from tobacco binds to microtubules and the plasma membrane. *Plant Cell* 13 2143–2158. 10.2307/3871433 11549769 PMC139457

[B14] Grbic V. Bleecker A. B. (1995). Ethylene regulates the timing of leaf senescence in *Arabidopsis.* *Plant J.* 8 595–602. 10.1046/j.1365-313X.1995.8040595.x

[B15] Guo Y. Gan S. (2005). Leaf senescence: signals, execution, and regulation. *Curr. Top. Dev. Biol.* 71 83–112. 10.1016/S0070-2153(05)71003-6 16344103

[B16] Halevy A. H. Porat R. Spiegelstein H. Borochov A. Botha L. Whitehead C. S. (1996). Short-chain saturated fatty acids in the regulation of pollination-induced ethylene sensitivity of Phalaenopsis flowers. *Physiol. Plant.* 97 469–474. 10.1111/j.1399-3054.1996.tb00505.x

[B17] Hazel J. R. Williams E. E. (1990). The role of alterations in membrane lipid-composition in enabling physiological adaptation of organisms to their physical-environment. *Progr. Lipid Res.* 29 167–227. 10.1016/0163-7827(90)90002-3 2131463

[B18] He Y. H. Gan S. S. (2002). A gene encoding an acyl hydrolase is involved in leaf senescence in *Arabidopsis*. *Plant Cell* 14 805–815. 10.1105/tpc.010422 11971136 PMC150683

[B19] Jia Y. Tao F. Li W. (2013). Lipid profiling demonstrates that suppressing *Arabidopsis* phospholipase Dδ retards ABA-promoted leaf senescence by attenuating lipid degradation. *PLoS ONE* 8:e65687 10.1371/journal.pone.0065687 PMC3676348 23762411

[B20] Keech O. Pesquet E. Gutierrez L. Ahad A. Bellini C. Smith S. M. (2010). Leaf Senescence is accompanied by an early disruption of the microtubule network in *Arabidopsis*. *Plant Physiol.* 154 1710–1720. 10.1104/pp.110.163402 20966154 PMC2996031

[B21] Li M. Hong Y. Wang X. (2009). Phospholipase D- and phosphatidic acid-mediated signaling in plants. *Biochim. Biophys. Acta* 1791 927–935. 10.1016/j.bbalip.2009.02.017 19289179

[B22] Li W. Li M. Zhang W. Welti R. Wang X. (2004). The plasma membrane-bound phospholipase D delta enhances freezing tolerance in *Arabidopsis thaliana*. *Nat. Biotechnol.* 22 427–433. 10.1038/nbt949 15004566

[B23] Li W. Wang R. Li M. Li L. Wang C. Welti R. (2008). Differential degradation of extraplastidic and plastidic lipids during freezing and post-freezing recovery in *Arabidopsis thaliana*. *J. Biol. Chem.* 283 461–468. 10.1074/jbc.M706692200 17962199

[B24] Lim P. O. Kim H. J. Nam H. G. (2007). Leaf senescence. *Annu. Rev. Plant Biol.* 58 115–136. 10.1146/annurev.arplant.57.032905.105316 17177638

[B25] Marechal E. Block M. A. Dorne A. J. Joyard J. (1997). Lipid synthesis and metabolism in the plastid envelope. *Physiol. Plant.* 100 65–77. 10.1034/j.1399-3054.1997.1000106.x

[B26] Mishra S. Shanker S. Sangwan R. S. (1998). Lipid profile in relation to tropane alkaloid production and accumulation during leaf growth and senescence in *Duboisia myoporoides*. *Fitoterapia* 69 65–72.

[B27] Munnik T. (2001). Phosphatidic acid: an emerging plant lipid second messenger. *Trends Plant Sci.* 6 227–233. 10.1016/S1360-1385(01)01918-5 11335176

[B28] Munnik T. Musgrave A. (2001). Phospholipid signaling in plants: holding On to Phospholipase D. *Sci. Signal.* 2001:pe42. 10.1126/stke.2001.111.pe42 11734658

[B29] Qin C. Wang X. (2002). The *Arabidopsis* phospholipase D family. Characterization of a calcium-independent and phosphatidylcholine-selective PLD zeta 1 with distinct regulatory domains. *Plant physiol.* 128 1057–1068. 11891260 10.1104/pp.010928 PMC152217

[B30] Rea G. de Pinto M. C. Tavazza R. Biondi S. Gobbi V. Ferrante P. (2004). Ectopic expression of maize polyamine oxidase and pea copper amine oxidase in the cell wall of tobacco plants. *Plant Physiol.* 134 1414–1426. 10.1104/pp.103.036764 15064377 PMC419818

[B31] Siefermann-Harms D. Ninnemann H. Yamamoto H. Y. (1987). Reassembly of solubilized chlorophyll-protein complexes in proteolipid particles — Comparison of monogalactosyldiacylglycerol and two phospholipids. *Biochim. Biophys. Acta Bioenerget.* 892 303–313. 10.1016/0005-2728(87)90234-9

[B32] Singh H. Privett O. S. (1970). Studies on the glycolipids and phospholipids of immature soybeans. *Lipids* 5 692–697. 10.1007/BF02531436 4318123

[B33] Suttle J. C. Kende H. (1980). Ethylene action and loss of membrane integrity during petal senescence in Tradescantia. *Plant Physiol.* 65 1067–1072. 10.1104/pp.65.6.1067 16661332 PMC440482

[B34] Thompson J. Taylor C. Wang T. W. (2000). Altered membrane lipase expression delays leaf senescence. *Biochem. Soc. Trans.* 28 775–777. 10.1042/bst0280775 11171204

[B35] Thompson J. E. Froese C. D. Madey E. Smith M. D. Hong Y. (1998). Lipid metabolism during plant senescence. *Prog. Lipid Res.* 37 119–141. 10.1016/S0163-7827(98)00006-X 9829123

[B36] Thompson J. E. Mayak S. Shinitzky M. Halevy A. H. (1982). Acceleration of membrane senescence in cut carnation flowers by treatment with ethylene. *Plant Physiol.* 69 859–863. 10.1104/pp.69.4.859 16662309 PMC426318

[B37] Wang C. Wang X. (2001). A novel phospholipase D of *Arabidopsis* that is activated by oleic acid and associated with the plasma membrane. *Plant Physiol.* 127 1102–1112. 10.1104/pp.010444 11706190 PMC129279

[B38] Wanner L. Keller F. Matile P. (1991). Metabolism of radiolabeled galactolipids in senescent barley leaves. *Plant Sci.* 78 199–206. 10.1016/0168-9452(91)90199-I

[B39] Welti R. Li W. Li M. Sang Y. Biesiada H. Zhou H. E. (2002). Profiling membrane lipids in plant stress responses. Role of phospholipase D alpha in freezing-induced lipid changes in *Arabidopsis*. *J. Biol. Chem.* 277 31994–32002. 12077151 10.1074/jbc.M205375200

[B40] Woolhouse H. W. (1984). The biochemistry and regulation of senescence in chloroplasts. *Can. J. Bot.* 62 2934–2942. 10.1139/b84-392

[B41] Zhang Q. Lin F. Mao T. Nie J. Yan M. Yuan M. (2012). Phosphatidic acid regulates microtubule organization by interacting with MAP65-1 in response to salt stress in *Arabidopsis*. *Plant Cell* 24 4555–4576. 10.1105/tpc.112.104182 23150630 PMC3531852

[B42] Zhang W. Wang C. Qin C. Wood T. Olafsdottir G. Welti R. (2003). The oleate-stimulated phospholipase D, PLDδ, and phosphatidic acid decrease H2O2-induced cell death in *Arabidopsis. Plant Cell* 15 2285–2295. 14508007 10.1105/tpc.013961 PMC197295

